# Long Noncoding RNA UCA1 Is Related to Autophagy and Apoptosis in Endometrial Stromal Cells

**DOI:** 10.3389/fonc.2020.618472

**Published:** 2021-02-18

**Authors:** Lili Jiang, Yahui Wan, Ziyi Feng, Da Liu, Ling Ouyang, Yan Li, Kuiran Liu

**Affiliations:** ^1^ Department of Obstetrics and Gynecology, Shengjing Hospital of China Medical University, Shenyang, China; ^2^ College of Clinical Medicine Science, China Medical University, Shenyang, China

**Keywords:** urothelial carcinoma-associated 1, endometriosis, autophagy, apoptosis, light chain 3, vacuole membrane protein 1

## Abstract

**Research Question:**

The expression of the long noncoding RNA (lncRNA) urothelial carcinoma-associated 1 (UCA1) in embryonic tissues is higher than that in most cancer tissues, such as bladder cancer, indicating that RNA is a carcinoembryonic antigen. However, there are no published reports on the role of UCA1 in endometriosis (EMS). Therefore, to address this gap in knowledge, we assessed the potential role of lncRNA UCA1 in the pathogenesis and progression of EMS.

**Design:**

To verify the expression of UCA1 in EMS, quantitative reverse transcription polymerase chain reaction (qRT-PCR) was used. RNA interference (siRNA) was used to study the biological function of UCA1 in EMS *in vitro*.

**Results:**

qRT-PCR analysis showed that the expression of lncRNA UCA1 in EMS was increased (P<0.01). Knockdown of UCA1 *in vitro* significantly inhibited the proliferation of endometrial stromal cells (ESCs) and induced autophagy and apoptosis.

**Conclusion:**

UCA1 is highly expressed in EMS and promotes the proliferation of ESCs but suppresses autophagy and apoptosis. In EMS, UCA1 may be a prognostic marker and therapeutic target.

## Introduction

Endometriosis (EMS) is a common benign gynecological disease that is estrogen-dependent. The growth and proliferation of endometrial tissues or cells outside the uterus, most commonly in the ovaries and pelvic peritoneum, are characteristic of EMS. In reproductive age, 10%–15% women are affected by endometriosis. Under the influence of the disease, 70% of the patients suffer from chronic pelvic pain and 48% have fertility problems (1). The main clinical complications of this disease include severe menstrual, several nonmenstrual pain and subfertility (1). The most generally acknowledged hypothesis about the pathogenesis of EMS is the implantation and growth of disseminated endometrial fragments and viable cells after retrograde flow of menses during menstruation (2). Although menstrual reflux is accepted, it does explain why 90% of women experience menstrual retrograde but that only 10% develop the disease (3). The pathogenesis of EMS remains uncertain. The identification of novel treatments and new prognostic indicators is needed.

Long noncoding RNAs (lncRNAs) are more than 200 nucleotides long and do not have protein-coding functions but participate in significant biological activities (4). Research on lncRNAs has initially revealed the etiology and pathogenesis of certain tumors or other diseases. At present, more than ten lncRNAs related to autophagy have been found. However, the biological role of lncRNAs in EMS remains unclear.

As a type of lncRNA, urothelial carcinoma-associated 1 (UCA1) was first identified in bladder transitional cell carcinoma (5). UCA1 is highly expressed in numerous cancers, including non-small cell lung cancer, gastric cancer and ovarian cancer. UCA1 was found to be a very sensitive and specific tumor-specific marker. It can play an important role in tumor diagnosis, treatment, prognosis, and postoperative non-invasive follow-up, suggesting that UCA1 may play a key role in human cancers or other diseases (6–9).

Endometriosis is still a major challenge for reproductive health due to multiple causes, coupled with the heterogeneity of the disease and the lack of appropriate diagnostic markers and treatment methods (10). Previous studies have shown that EMs is associated with changes in the expression of long non-coding RNAs (lncRNAs). Yu et al. (11) have found that lncRNA MALAT1 can promote endometrial cell apoptosis and regulate the expression of MMP-9 through the NF-κB/iNOS pathway, thereby mediating the pathogenesis of EMs. Other studies have found that LncRNA BANCR can inhibit the development of ectopic endometrium by repressing the formation of angiogenesis factors in EMs (12). It is concluded that lncRNAs is related to the occurrence and development of EMs, and may play a key role in prognostic, diagnostic or therapeutic role in EMs. In this study, we discovered that UCA1 was overexpressed in EMS. We studied the effect of UCA1 on the proliferation, autophagy and apoptosis of ESCs *in vitro*. This is the rare report investigating the role of UCA1 in EMS.

## Materials and Methods

### Tissue Collection

All patients were from Shengjing Hospital, China Medical University. First, 30 surgical patients (23–51 years old) with EMS and 30 surgical patients (22–50 years old) with cervical intraepithelial neoplasia (CIN) were selected to obtain endometrial tissue specimens. There was no significant difference in pregnancy, birth or age between the two groups. None of these patients received any hormonal drugs or GnRH analog within the 6 months before the surgery. The patients didn’t implant intrauterine devices. Those with other diseases of the reproductive system, as well as those with severe organ failure, malignant tumors or complications were excluded. The clinical characteristics of the patients of the two groups shown in **Table 1**. Fresh, sterile specimens were frozen in liquid nitrogen and stored at –80°C. After our conclusion was in line with the forecast, fifteen patients (28–47 years old) without birth requirements with stage III-IV ovarian EMS (according to the revised American Fertility Society classification system) undergoing total hysterectomy were selected for the subsequent study. All diagnoses were pathologically confirmed. The collection of specimens was authorized by the patient herself or her family. The specimens collected for use in this study were approved by the China Medical University Research Ethics Committee.

**Table 1:** Clinical characteristics of the patients in EMS group and normal endometrium group.

Characteristics	EMs group (n=30)	Normal endometrium group (n=30)	t/χ^2^	P Value
Age	36.63 ± 7.87	38.87 ± 7.96	-1.093	0.279
Gravidity	2.06 ± 0.89	2.49 ± 1.09	-1.365	0.178
Parity	1.20 ± 0.61	1.53 ± 0.78	-1.849	0.07
Menstruation				
None-Menopause	28	26	0.741	0.389
Menopause	2	4		

### Cell Isolation and Culture

Endometrial stromal cell (ESC) cultures were isolated from endometrial tissues according to the following protocol. In brief, endometrial tissue samples were cut into small pieces and digested in 0.1% collagenase (type I) (Sigma-Aldrich, Shanghai, China) for 60 min with a shaking water bath at 37°C. The digested cells were passed through a nylon sieve (aperture of 38 μm), and the eluate containing the stromal cells was then centrifuged at 1000 r for 10 min at room temperature. The obtained cell pellet was washed once and resuspended in DMEM/F12 (HyClone, Logan, Utah, USA) supplemented with 10% fetal bovine serum (FBS; Gemini, Calabasas; CA, USA) in a sterile incubator at 37°C and 5% CO_2_. The purity of the cell cultures was higher than 99% after the third passage as determined by analysis of cellular markers *via* immunocytochemical staining methods (all antibodies were from Zhongshan Goldenbridge, Beijing, China). Cells from passage 3 to passage 5 were used in these experiments.

### Isolation of Total RNA, Reverse Transcription, and Quantitative Reverse Transcription Polymerase Chain Reaction

TRIzol reagent (9108, Invitrogen, Carlsbad, CA, USA) was used to isolate total RNA from tissues and cells. All steps were performed without RNase. RNA optical density (OD) and concentration were read by a UV spectrophotometer (Bio-Rad, California, USA). A PrimeScript RT reagent kit (RR047A, TaKaRa Bio Inc., Japan) was used to perform the reverse transcription reactions. A SYBR PremixEx TaKaRa kit (RR420A, TaKaRa Bio Inc., Japan) and an ABI 7500 fast real-time system (Life Technologies, Foster City, CA, USA) were used for qRT-PCR. All steps of qRT-PCR strictly followed the manufacturers’ protocols.

Relative lncRNA mRNA expression levels (mean ± SD) were calculated using the 2^-△△Ct^ method with glyceraldehyde-3-phosphate dehydrogenase (GAPDH) as the endogenous control. Each sample was assayed in triplicate. The qRT-PCR primers were designed by Primer Premier 5.0 software (Premier, Canada), and their sequences are shown in **Table 2**.

**Table 2:** Primer sequences used in qRT-PCR.

RNA	Primers
UCA1	Forward: 5’-TTCCACATATTTGGCAACCAGAC-3’
	Reverse: 5’-GATTAAGCTGAGGCTGGCAAAG-3’
LC3	Forward: 5’-AACATGAGCGAGTTGGTCAAG-3’
	Reverse: 5’-GCTCGTAGATGTCCGCGAT-3’
VMP1	Forward: 5’- TATGCCAAACGAATCCAGCAG -3’
	Reverse: 5’- CCAGTCTGTTGCAAGTTTGCTG -3’
GAPDH	Forward: 5’-GCACCGTCAAGGCTGAGAAC-3’
	Reverse: 5’-ATGGTGGTGAAGACGCCAGT-3’

TRIzol reagent (9108, Invitrogen, Carlsbad, CA, USA) was used to isolate total RNA from tissues and cells. All steps were performed without RNase. RNA optical density (OD) and concentration were read by a UV spectrophotometer (Bio-Rad, California, USA). One microgram of RNA was reverse transcribed using the Prime Script RT reagent kit (RR047A, TaKaRa Bio Inc., Japan). The QuantiTect SYBR Green RT-PCR Kit (QIAGEN) was used to prepare the qPCR reaction. PCRs were performed on an ABI PRISM 7500 sequence detection system (Applied Biosystems; Thermo Fisher Scientific, Inc.). For the measurement of miRNA expression, miRNA-specific cDNA was synthesized from 5 ng of total RNA using the TaqMan MicroRNA Reverse Transcription Kit (Applied Biosystems) on an ABI PRISM 7500 sequence detection system (Applied Biosystems). The expression levels of U6 small nuclear RNA and β-actin mRNA were used as reference genes. All PCRs were repeated 3 times, and the mean values are presented. Fold changes in gene expression were measured using the 2^−ΔΔCT^ method. The primer sequences are shown in **Table 2**.

### Lentiviral Transfection

LncRNA UCA1 siRNA (si-UCA1) and a negative control (siRNA-NC) were purchased from GeneChem (Shanghai, China). The sequences of these siRNAs are shown in **Table 3**. Both siRNAs were transfected into cells cultured in 6-well plates following the manufacturer’s instructions. Our study had the following two controls: 1) cells transfected with siRNA negative control (siRNA-NC); and 2) cells with no siRNA transfection, which was also called the mock control (Control). As recommended by the manufacturer’s instructions, the knockdown effect of si-UCA1 was assayed by qRT-PCR after transfection for 72 h. Every patient had individual cultures.

**Table 3:** siRNA sequences.

siRNA	Target Seq
si-UCA1	CTCCTGGAAGCCACAAGATTA
siRNA-NC	TTCTCCGAACGTGTCACGT

### Cell Proliferation Assay

Cell proliferation was measured using the Cell Counting Kit-8 assay (CCK-8; Dojindo Molecular Technologies, Osaka, Japan). Briefly, ESCs were seeded into 96-well plates and transfected with siRNA. After culturing for 0, 24, 48, and 72 h, 10 μl of CCK-8 reagent was added to all wells at 37°C for 1 h. Cells were then incubated for an additional 4 h. The absorbance at 450 nm of each well was measured using a microplate reader (Bio-Rad, Hercules, CA, USA). The cell proliferation rate was calculated by the ratio of the OD values of the experimental and control groups.

### Cell Lysosomal Fluorescence Intensity Assay

Cell lysosomal fluorescence intensity was estimated with a LysoTracker Red kit (Beyotime Biotechnology, Beijing, China). Higher fluorescence intensity represents stronger lysosomal activity. LysoTracker Red was added to each well, and the plates were incubated at 37°C for 2 h according to the manufacturer’s protocol. LysoTracker Red was then removed, and fresh medium was added. A fluorescence microscope (Nikon Eclipse NI microscope, Nikon, Inc., Japan) was used for imaging. Lysosomes showed bright fluorescence staining, and the results were analyzed by ImageJ analysis software.

### Cell Apoptosis Assay

An annexin V FITC apoptosis detection kit (Annexin FITC, Dojindo Molecular Technologies, Osaka, Japan kit) was used to analyze cell apoptosis. Cells were washed with 1x PBS and then diluted to 1x10^6^ cells/ml in 1× binding buffer. Subsequently, 10 μl of annexin V and PI (1:1) was added to 100 μl of the cell suspension. After incubation for an additional 15 min in the dark, 400 μl of 1× binding buffer was added to the media. FACS Calibur (Becton, Dickinson and Company, Franklin Lake, New Jersey, USA) analysis was used to detect and quantify positive cells.

### Western Blot Assay

Western blotting was performed as previously described (13). Anti-VMP1 was purchased from Abcam (Cambridge, Massachusetts, USA). Anti-LC3, anti-GAPDH and anti-cleaved PARP were purchased from ProteinTech Group (Wuhan, China). GAPDH was used as the control. The individual LC3 and VMP1 band intensities were then normalized to the corresponding GAPDH absorbance value. The band intensity was visualized and analyzed by an imager (Bio-Rad, Shanghai, China) after digitization with a scanner (Bio-Rad, CA, USA). The optical densities of the protein bands were measured using Image Lab software (Bio-Rad, Shanghai, China) after digitization with a scanner (Bio-Rad, CA, USA). GAPDH was used as the internal control. The band intensities of LC3 and VMP1 were then normalized to the corresponding GAPDH absorbance value.

### Statistical Analyses

All data was expressed as the mean ± SEM. SPSS 17.0 software (SSPS Inc., Chicago, USA) was used for the statistical tests. Statistical comparisons of means between groups were performed using paired Student’s t-test or ANOVA (one-way) followed by Tukey’s test. P < 0.05 was considered statistically significant.

## Results

### Expression of lncRNA UCA1 in EMS Tissue

**Figure 1:**
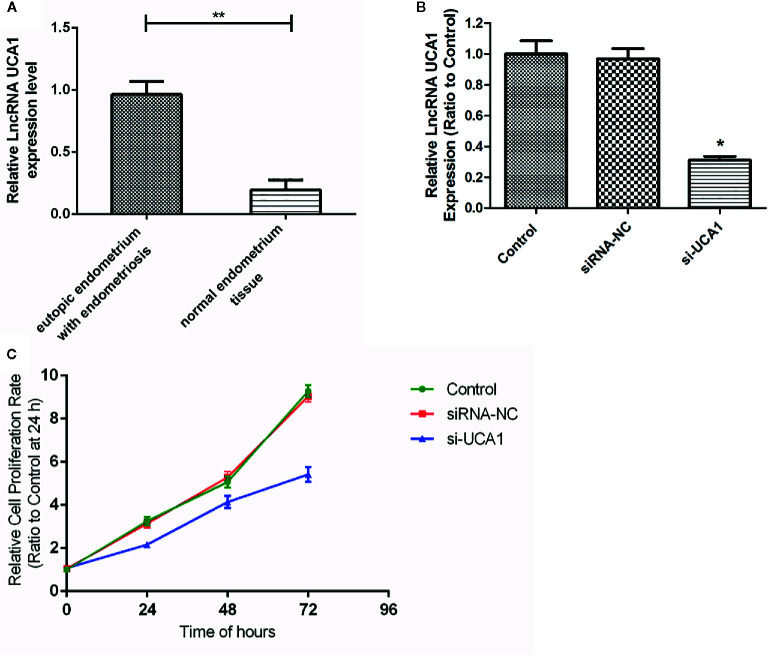
Expression of lncRNA UCA1 in EMS tissue. **(A)** The expression level of lncRNA UCA1 in the eutopic endometrium of patients with endometriosis was higher than that in normal endometrial tissues by qRT-PCR (*P<0.01). The interference effect of si-UCA1in ESCs. **(B)** Compared with the control and siRNA-NC groups, the expression levels of UCA1 in ESCs transfected with si-UCA1 were significantly decreased (*P<0.05). Effect of UCA1 on the proliferation of ESCs *in vitro*. **(C)** The proliferation abilities of cells decreased significantly after transfection with si-UCA1 compared to the siRNA-NC and control groups (*P<0.05, **P<0.01).

qRT-PCR revealed that the expression of lncRNA UCA1 was significantly higher in the eutopic endometrium of patients with endometriosis (0.965 ± 0.105) than in normal endometrial tissues (0.196 ± 0.079) (P<0.01; **Figure 1A**).

### Interference Effect of UCA1 siRNA in ESCs

After 72 h of siRNA transfection, the expression of UCA1 in ESCs was detected by qRT-PCR. Compared with control cells and siRNA-NC-transfected cells, UCA1 expression in ESCs transfected with si-UCA1 was decreased (P<0.01; **Figure 1B**).

### Effect of UCA1 on the Proliferation of ESCs *In Vitro*

The CCK-8 assay was used to determine whether lncRNA UCA1 affects the proliferation of ESCs *in vitro*. Compared with siRNA NC-transfected cells and control cells, the proliferation of ESCs decreased significantly after si-UCA1 transfection (P<0.05; **Figure 1C**; **Table 4**).

**Table 4:** Changes in cell proliferation after transfection with si-UCA1 compared with siRNA-NC and control cells.

Times	Control	siRNA-NC	si-UCA1	P Value (Control vs. siUCA1)	P Value (Control vs. siRNA-NC)
0 h	1.00 ± 0.09	1.05 ± 0.09	1.08 ± 0.09	–	–
24 h	3.25 ± 0.19	3.14 ± 0.21	2.16 ± 0.12	0.001*	0.6964
48 h	5.06 ± 0.26	5.29 ± 0.26	4.14 ± 0.28	0.001*	0.5343
72 h	9.28 ± 0.27	9.04 ± 0.25	5.42 ± 0.34	0.001*	0.5263

### Effect of UCA1 on the Lysosomal Fluorescence Intensity of ESCs *In Vitro*

**Figure 2:**
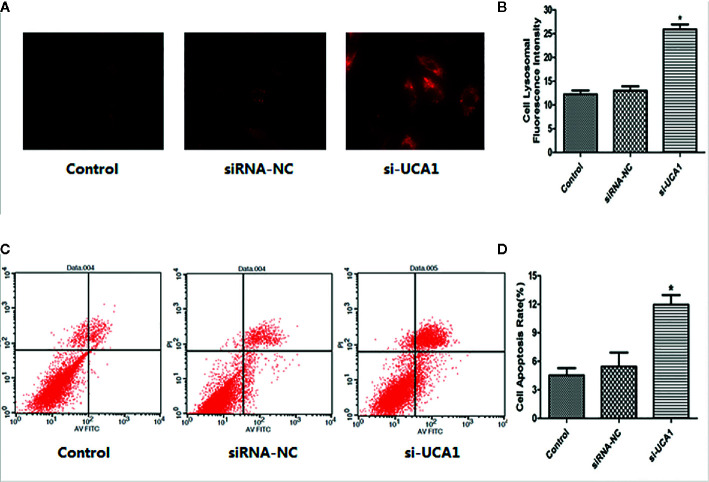
Effect of UCA1 on ESC autophagy and apoptosis *in vitro*. **(A, B)** In the cell lysosomal fluorescence intensity assay, the cell lysosomal fluorescence intensity was significantly increased after transfection with si-UCA1 compared to the siRNA-NC and control groups (P*<0.05). **(C, D)** In the cell apoptosis assay, the apoptosis ability of cells was significantly increased with si-UCA1 transfection compared to the control and siRNA-NC groups (*P<0.05).

A fluorescent staining assay was used to determine whether lncRNA UCA1 affects the lysosomal activity of ESCs *in vitro*. Compared with the control and siRNA-NC groups, the lysosomal fluorescence intensity of ESCs was significantly higher after transfection with si-UCA1 (P<0.05; **Figures 2A, B**).

### Effect of UCA1 on ESC Apoptosis *In Vitro*

The FACS assay was used to determine whether lncRNA UCA1 affects the apoptosis of ESCs *in vitro*. Compared with control and siRNA-NC-transfected cells, the apoptosis rate of ESCs was significantly increased after si-UCA1 transfection (P<0.05; **Figures 2C, D**).

### Effect of UCA1 on LC3 and VMP1 Expression

**Figure 3:**
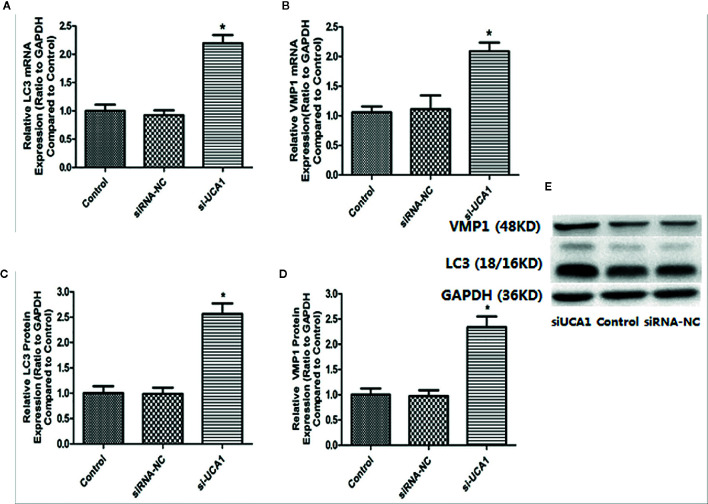
**(A, B)** In the si-UCA1 group, LC3 and VMP1 mRNA levels were significantly increased compared with those in the control and siRNA-NC groups (P<0.05). **(C, D)** Compared to the control and siRNA-NC groups, LC3 and VMP1 protein expression levels were significantly increased in cells transfected with si-UCA1 (P<0.05). **(E)** LC3 and VMP1 protein levels after si-UCA1 transfection.

LC3 is a marker protein on the autophagosome membrane. Since LC3 is needed during the formation of the autophagosome membrane, the autophagosome can be identified by labeling LC3. VMP1, as an autophagy-related gene that has been identified, can also participate in the autophagy process of cells. So, the mRNA and protein expression levels of LC3 and Vmp1 after transfection with si-UCA1 were detected by qRT-PCR and Western blot analyses. Compared with control and siRNA-NC cells, the expression of LC3 and VMP1 mRNA (**Figures 3A, B**; **Table 5**) and protein (**Figures 3C–E**; **Table 5**) in si-UCA1 cells was significantly increased (P<0.05). These results indicated that lncRNA UCA1 may at least partially activate the signaling pathway directly and then regulate the expression of LC3/VMP1 to promote cell proliferation, migration and invasion.

**Table 5:** LC3 and VMP1 mRNA and protein levels in si-UCA1-transfected cells compared to the control and siRNA-NC groups.

Target gene	Relative expression level compared to Control			P Value(Control vs. siRNA-NC)	P Value(Control vs. si-UCA1)
	Control	siRNA-NC	si-UCA1		
LC3 mRNA	1.00 ± 0.11	0.92 ± 0.08	2.20 ± 0.14	0.5924	0.001*
LC3 protein	1.00 ± 0.14	0.99 ± 0.12	2.57 ± 0.21	0.9438	0.001*
VMP1 mRNA	1.00 ± 0.10	1.11 ± 0.23	2.09 ± 0.15	0.8418	0.001*
VMP1 protein	1.00 ± 0.12	0.97 ± 0.11	2.34 ± 0.21	0.8773	0.001*

## Discussion

As a gynecological disorder, endometriosis is the disease which the main clinical complications include severe menstrual and non-menstrual pain and subfertility (14). The most common hypothesis about the etiology of EMS is retrograde menstruation. However, implant or persistent deposition of lesions in the pelvic cavity is another reason that can be influenced by inflammatory, hormonal and immunologic environments (1). The diagnosis of endometriosis is inadequate and usually delayed. The incubation period from the onset of symptoms to a clear diagnosis is longer. Among patients 18–45 years old, the average delay time is 6.7 years, and some patients require surgical diagnosis (15). Endometriosis has biological characteristics similar to those of malignant tumors with a variety of pathological morphologies and invasion, which seriously affects the patient’s quality of life. Therefore, it is of great significance to study the pathogenesis of endometriosis, which may contribute to the prevention, diagnosis, treatment and improvement of the prognosis of the disease.

It is estimated that 70% to 90% of transcription results in long noncoding RNAs in mammalian genomes (16). In the epigenetic state of the human genome, long noncoding RNAs are important regulators. In addition to their involvement in normal physiology, the expression and function of lncRNAs have been proven to be related to many diseases, including cancer (17). The occurrence of endometriosis has similar clinical characteristics to malignant tumors. Whether the pathogenesis is similar to that of malignant tumors has become a research direction of endometriosis etiology. A total of 1277 lncRNAs (789 downregulated and 488 upregulated) and 1216 mRNAs (638 downregulated and 578 upregulated) between eutopic and normal endometrium were differentially expressed by microarray expression profiling. Differentially expressed lncRNAs have been found to be associated with immune regulation and the cell cycle by gene ontology analysis and pathway analysis (18). As a types of lncRNA, UCA1, which is located on human chromosome 19p13.12, has been widely confirmed in a variety of tumors due to its carcinogenic effect (19). C/EBPα and Ets-2 are the transcription factors which can regulate the expression of UCA1, and the cancer progression can be promoted by UCA1 overexpression through different pathways, including PI3K, AKT, and mTOR-STAT3 signal pathways (20–22). UCA1 can also interact with microRNAs(miRNAs), a type of regulatory ncRNA to compete endogenous RNA in cancer cells. UCA1 promotes the development of tumors by regulating cell proliferation (23–25), invasion (24, 25), migration (25), metastasis (26), apoptosis (27), metabolism (22), survival (24), radiosensitivity and chemoresistance (28, 29). Thus, we have focused on whether UCA1 has significance in the study of endometriosis.

In this study, qRT-PCR analysis showed that the expression level of UCA1 in the eutopic endometrium of patients with endometriosis was significantly higher than that in the normal endometrium. The results demonstrated that high expression of UCA1 might accelerate the progression of EMS, which is consistent with its changes in various malignant tumors. Our results suggested that UCA1 might be involved in the occurrence and development of EMS by promoting cell proliferation and inhibiting apoptosis.

As a disease with growth characteristics, cell proliferation and apoptosis play important and indispensable roles in the development of EMS. For normal development and tissue size homeostasis, the coordination and balance between cell proliferation and apoptosis are crucial (30) as disease occurs once the balance is broken. Apoptosis is a unique form of programmed cell death defined by characteristic morphological and biochemical events that can lead to the effective removal of cells from tissues without causing inflammation (31). Apoptosis is a rational and positive process that can fight the defense mechanism of pathogens and is essential for embryogenesis and cell homeostasis. Previous studies have indicated that apoptosis indices of eutopic endometrium in patients with EMS are lower than those in women without EMS (32). Endometrial cells are fundamentally different in women with and without EMS, which has been verified by increasing evidence. The proliferation of endometrial cells in patients with EMS is enhanced, and the implantation ability and survival in ectopic sites are increased. The decreased sensitivity of endometrial tissue to spontaneous apoptosis leads to abnormal implantation and growth of ectopic endometrium. Apoptosis may play an important role in the development of EMS (33).

Autophagy is a closely coordinated process that delivers cellular material to lysosomes for degradation and plays an important role in maintaining cell homeostasis (34). New research has shown that autophagy is closely related to EMS. However, autophagy may be upregulated or downregulated in EMS through different signaling pathways. Thus, it remains unknown if autophagy is a foe or friend in EMS (35). Further research on the exact role of autophagy in EMS, especially *in vivo* studies and the detection of more autophagy-related proteins, has become particularly important.

Several autophagy-related genes (ATGs), including microtubule-associated light chain 3 (LC3) and beclin-1, play important roles in autophagy and are often regarded as potential biomarkers of autophagy (36). In different stages of autophagy, Beclin-1 and LC3 play a role. Although Beclin-1 is considered to be an important part of autophagy initiation, LC3 is involved in the later stages, and LC3-II is a marker of autophagy (36). A previous study has reported that both LC3 and LysoTracker are upregulated in different biological events during autophagy (37).

Specific genes known as ATGs regulate autophagy. Many ATG proteins, including vacuole membrane protein 1 (Vmp1), have been identified. VMP1 interacts with Beclin-1, which positively regulates the formation of autophagosomes (38). In addition, it is known that autophagy can inhibit apoptosis and that apoptosis can regulate autophagy, but the detailed mechanism of these two pathways is still not clear in ESCs (39).

We can use high-throughput microarray to identify and screen specific lncRNAs, the new markers of some cancers. RNA interference mediated selective silencing of lncRNA may be a therapeutic option for some cancers. In this study, we found that the expression of UCA1 in the eutopic endometrium of patients with endometriosis was higher than that in the normal endometrium by qRT-PCR. In vivo, the expression levels of UCA1 in ESCs and the proliferation abilities of cells decreased significantly after knockout of lncRNA UCA1. The results show that UCA1 may promote cell proliferation of EMS. After si-UCA1 transfection, the apoptosis ability of ESCs was significantly enhanced by flow cytometry. LC3 is a marker protein on the autophagosome membrane. Since LC3 is needed during the formation of the autophagosome membrane, the autophagosome can be identified by labeling LC3. VMP1, as an autophagy-related gene that has been identified, can also participate in the autophagy process of cells. So we detected the mRNA and protein levels of LC3 and VMP1 and found that were significantly increased after UCA1 knockout, which further demonstrated that UCA1 may inhibit the autophagy of ESCs. These results indicated that UCA1 has a significant effect on the proliferation, apoptosis and autophagy of ESCs. The strong proliferation ability and the decreased autophagy and apoptosis increase the ectopic survival ability of endometrial cells and cause the occurrence of EMS. The role of UCA1 in the pathogenesis of endometriosis needs further study.

In conclusion, lncRNA-UCA1 is highly expressed in the eutopic endometrium of patients with endometriosis. Knockout of the UCA1 gene inhibits the proliferation of ESCs, promotes apoptosis, promotes autophagy and participates in the occurrence and development of endometriosis. Future animal research and bioinformatics evaluation will help to determine the mechanism of UCA1 in endometriosis.

## Data Availability Statement

The original contributions presented in the study are included in the article, further inquiries can be directed to the corresponding author/s.

## Ethics Statement

The studies involving human participants were reviewed and approved by Medical Ethics Committee of Shengjing Hospital Affiliated to China Medical University. The patients/participants provided their written informed consent to participate in this study.

## Author Contributions

LJ conducted main part of the experiment and was the major contributor in writing the manuscript. YW was responsible for part of the literature search and experiment. ZF and DL collected specimens and took the images. LO provided topic support and experiment consultation. YL was responsible for drawing and reviewing pictures. KL was responsible for checking the data, reviewing, and revising the article. All authors contributed to the article and approved the submitted version.

## Funding

This research received the support from “Scientific research funding project of Liaoning Provincial Department of Science and Technology (No.2020JH2/10300050)”, “Research funding project of Liaoning Provincial Department of Education (No.JC2019012)”, and “Shenyang Science and Technology Planning Project (No.19-112-4-020)”.

## Conflict of Interest

The authors declare that the research was conducted in the absence of any commercial or financial relationships that could be construed as a potential conflict of interest.
